# Critical limits for early detection of glaucoma, the Uppsala Glaucoma Detection Study (UGDS)

**DOI:** 10.1007/s10792-026-03949-4

**Published:** 2026-01-29

**Authors:** Konstancija Kisonaite, Tolga Tümer, Albert Alm, Eva Nuija, Zhaohua Yu

**Affiliations:** Gullstrand lab, Ophthalmology, Department of Surgical Sciences, Uppsala University, SE-751 85 Uppsala, Sweden

**Keywords:** Glaucoma, Clinical evaluation, Specificity, Critical limits, Variability, Tolerance limits, IOP, MD, C/D-linear, NRA, RNFL, TSNIT

## Abstract

**Purpose:**

The current study aimed to optimize the measurement design for each quantity of interest, determine critical limits for clinically distinguishing glaucoma-suspect eyes from non-suspect eyes in the UGDS using the optimized design, and compare the efficiency of the measured quantities in estimating sensitivity

**Methods:**

Data from non-glaucoma suspect eyes in the UGDS were analyzed for age and sex dependence, sources of variation, frequency distribution, and assessment of critical limits for glaucoma detection. Critical limit was defined as the extreme 95% confidence limit for the 95% one-sided tolerance. Intraocular pressure (IOP) and visual field contract sensitivity (Mean Deviation, MD), linear cup-to-disc ratio (C/D-linear), neuro-retinal rim area (NRA), nerve fiber layer thickness (cpRNFLT-Global and GDx-TSNIT) were examined

**Results:**

Analysis revealed no significant age or sex dependence for the measured quantities. Variability among subjects was found to dominate, affecting the precision of measurements. Frequency distributions approximated normal distributions, enabling the estimation of tolerance limits for critical assessment. The critical limits to distinguish pathological from non-pathological were estimated as 22 mmHg, −3.7 dB, 0.84, 0.6 mm^2^, 56 µm and 33 µm for IOP, MD, C/D-linear, NRA, cpRNFLT-Global and GDx-TSNIT respectively

**Conclusion:**

It appears preferable to estimate critical limits for small samples using the extreme confidence limit of the tolerance limit. The critical limits obtained here are consistent with previously reported values for the same device models. C/D-linear and NRA-Global metrics estimated with HRT show lower sensitivity-estimation efficiency compared with the other parameters assessed. Individualized intra-patient critical limits require a small increase of the measured quantity to identify glaucoma in the patient who has the disease

## Introduction

Intraocular pressure (IOP), estimation of spatial distribution of the visual field, and later morphometric measurement of the optic nerve head were adopted for glaucoma detection. Development of computerized visual field estimation allows quantitative estimation of spatially resolved perceived sensitivity in the visual field [15]. Considerable efforts have been made to make the measurements efficient and to summarize the measurements in clinically meaningful variables [4], [6], Heijl, Lindgren & Olsson 1987).

Measurement precision limits the possibility to differentiate a pathological measurement from not pathological. For this reason, it is desirable that iterated measurements at the same occasion can be averaged. Despite substantial improvements [7], [18], computerized visual field estimation is still time consuming and bothersome for the patient and therefore not suitable for iterated measurements.

In recent years several technologies that allow 2D, and lately 3D capture of structures at the back of the eye have emerged. This development triggered several efforts to develop strategies to quantitatively measure the nerve fiber layer in the ONH or its vicinity for evaluation of glaucoma (Table 1).

**Table 1:** Quantities for measurements of the nerve fiber layer

Quantity	Unit	Abbreviation	Description
Neuro-retinal rim area	mm^2^	NRA	Frontal projection of the cross-sectional area of the nerve fiber layer in the ONH
Cup-to-disc ratio, linear	rel.	C/D-linear	Ratio of vertical cup diameter to vertical disc diameter
Nerve fiber layer thickness	µm	GDx-TSNIT	Shift of polarization angle associated with the retinal nerve fiber layer thickness
Circumpapillary retinal nerve fiber layer thickness	µm	cpRNFLT-Global	Nerve fiber layer thickness circularly averaged in the vicinity of the ONH

ONH imaging for morphometric is fast and uncomplicated for the patient. Thus, precision of the measurement at one occasion can be improved by averaging several measurements.

Due to variation in level among not glaucoma suspect eyes it is necessary to define a critical limit for not glaucoma suspect eye as the measurement level inside which there is a defined probability to find a not pathological measurement, the specificity. If measurements of a glaucoma associated quantity are normal distributed, the critical limit can be derived from the mean and the standard deviation of the population of not pathological not glaucoma suspect eyes as a one-sided tolerance limit (Appendix 1). If the mean and standard deviation are estimated from a limited sample of not glaucoma eyes, the uncertainty in the estimates results in uncertainty with regard to the estimated tolerance level. The uncertainty can be expressed as an extreme confidence limit for the one-sided tolerance limit [14]. The critical limit is then the extreme confidence limit.

Sensitivity is defined as the proportion of pathological measurements in a pathological cohort that are above the critical limit for not pathological. For a defined mean and standard deviation of pathological measurements, the sensitivity therefore becomes lower if the distance between the mean of not pathological measurements and the critical limit increases. The distance between the mean of not pathological measurements and the critical limit normalized to the mean therefore is a unitless relative measure of the efficiency of the quantity for estimation of sensitivity.

Several investigations aimed to establish critical limits for the quantities in focus in the current paper (Table 2).

**Table 2:** Critical limits for glaucoma detection

Quantity	Unit	Critical limit	References
IOP	mmHg	21^1^, 23^1^, 23^1^	[23], [30], [33], [2]
MD	dB	−2^3^	[16]
NRA	mm^2^	0.9^1^	[3], [19], [36]
C/D-linear	rel.	0.7^1^, 0.8^1^	[12], [21]
cpRNFLT-Global	µm	74^2^, 80^2^, 85^1^, 90^2^	[28], [5], [22], [24]
GDx-TSNIT	µm	45^2^	[26], [25]

IOP, intraocular pressure; MD, mean deviation; NRA, neuroretinal rim area; C/D-linear, linear cup–disc ratio; cpRNFLT-Global, circumpapillary RNFL thickness; GDx-TSNIT, temporal–superior–nasal–inferior–temporal average. Tolerance-limit cutoffs from the literature are listed for comparison with the present study’s tolerance-limit estimates.

^1^one sided 95 % tolerance limit based on mean and standard deviation estimated in very large samples.

^2^extreme 95 % confidence limit for one-sided 95 % tolerance limit [14]based on mean and standard deviation estimated from limited sample size.

^3^stated limit for definite progression av MD

The novelty of the present study is its derivation of critical limits from a clinically well-characterized cohort of subjects that were longitudinally followed. This approach allows to identify how inter-individual and intra-individual variability, respectively, impacts the critical limits for early glaucoma detection.

The aim of the present article was to optimize the design for measurement of each of the quantities of interest. Further, it was planned to estimate critical limits for clinical distinction of glaucoma suspect eyes from not glaucoma suspect eyes in the UGDS, when applying optimized measurement design. Finally, it was intended to compare efficiency for estimating sensitivity among the quantities measured.

## Materials and methods

### Subjects

The current study is a sub-analysis of data collected in the Uppsala Glaucoma Detection Study (UGDS). The UGDS was designed as a five year prospective longitudinal follow up of subjects with at least one glaucoma-suspect eye (Appendix 2). Measurement of several quantities considered associated with glaucoma was planned on both eyes every 4^th^ month. Ethical permission was granted by the Regional ethical review board in Uppsala (Dnr 2005:052). A total of 88 subjects were included.

In the current sub-analysis, data collected from contralateral eyes not meeting the criteria for glaucoma suspect (Appendix 2) at the base-line or the first 4-month follow up, were analyzed. The sub-analysis is based on the baseline- (first occasion) and the first 4-month follow-up (second occasion) measurements. Data originated from 40 subjects (median age: 67 years; range: 45–82 years; n-males/n-females: 20/20).

### Equipment

Equipment used and intended number of iterations at each measurement occasion are summarized in Table 3.

**Table 3:** Equipment used for measurement and intended number of iterated measurements at each measurement occasion

Quantity	Device	Quantity abreviaion	No of planned iterations
Intraocular pressure	Haag-Streit (Switzerland)	IOP	1
Visual field contrast sensitivity	Humphrey 24-2 SITA-Standard (Zeiss, Germany)	MD	1
Cup-to-disc ratio, linear	HRT-II (confocal topography, Heidelberg Engineering, Germany)	C/D-linear	≥ 3
Neuro-retinal rim area	HRT-II (confocal topography, Heidelberg Engineering, Germany)	NRA	≥ 3
Nerve fiber layer thickness averaged over the circumference of the ONH in the vicinity of the ONH	OCT (Carl Zeiss, Germany)	cpRNFLT-Global*	≥ 3
	Stratus (time domain): 3.4 mm RNFL scan diameter		
	Cirrus 4000 : 3.46 mm scan diameter		
	Cirrus 5000 : 3.46 mm scan diameter		
	GDx VCC (polarimetry, Laser Diagnostic Techologies Inc., USA)	GDx-TSNIT	≥ 3

^*^Corresponding to *Average RNFL* in Cirrus.

### Initial examination of data

For each quantity measured, all measurements were initially visualized as a function of eye on scatter plots. No outliers were identified.

### Study design

One not glaucoma suspect eye in each of 40 subjects was measured at baseline and at the first 4 month follow up visit. Measurements of all quantities (Table 3) were intended for all subjects at all visits. For several quantities measurements were iterated at each occasion (Table 3).

### Statistical analysis

The confidence coefficient was set to 0.95 considering the sample size. The one sided tolerance limit was set to 0.95. Matlab was used for all computations. Normality of each measurement was evaluated visually using Normal Equivalent Deviate (NED) plots [13]. The impact of age and sex was analyzed with multiple regression (Appendix 3). Variance components (among subjects, occasions within subjects, and iterations within occasion) were estimated using ANOVA (Appendix 4 and 5). Confidence limits for one sided tolerance limits were estimated according to [14] (Appendix 1). The efficiency of the quantity for estimation of sensitivity was estimated as distance between the critical limit and the mean of not pathological measurements normalized to the mean of not pathological measurements (rel.).

## Results

### Age and sex dependence in measurements in not glaucoma suspect eyes

**Fig. 1:**
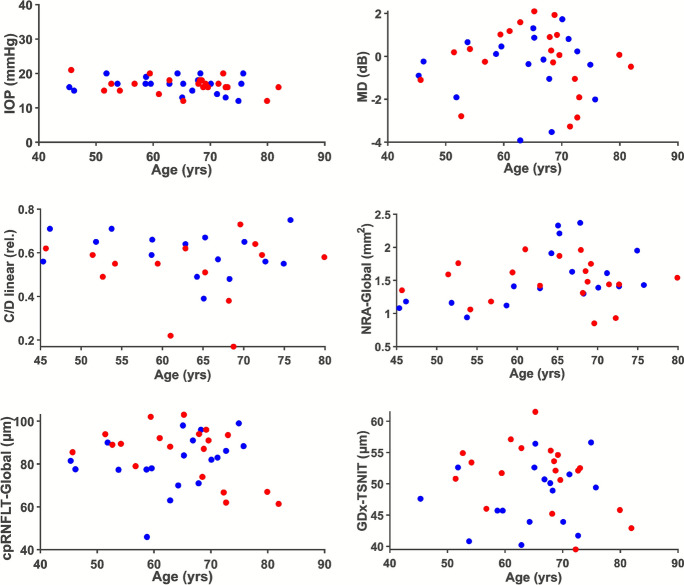
Analysis of dependence on age and sex in response variables. Male (blue) and female (red) primary measurements in glaucoma non-suspect eyes at baseline exam as a function of age

The first iteration of the first occasion was analyzed for age and sex dependence. There was no apparent dependence of any of the measured quantities on age or sex (Fig. 1).

The potential dependence of the quantity on age and sex at base line was analyzed by fitting the measurements from the first iteration on the first occasion according to the model in Appendix 1, Eq. 1. The regression analysis indicated no dependence of sex or interaction of age and sex for any of the response variables analyzed. Subsequent analysis of age dependence only, did not resolve age dependence (Table 4, CI_k_(0.95).

**Table 4:** Age dependence on response variables analyzed in glaucoma not suspect eyes

Quantity	Age at first exam	CI_k_(0.95)	D.f
IOP (mmHg)	[45;81]	−5.50 x10^−2^ ±8.70 x10^−2^ (mmHg·yrs^−1^)	39
MD (dB)	[45;81]	−1.80 x10^−3^ ±56.0 x10^−3^ (dB·yrs^−1^)	37
C/D-linear (rel.)	[45;79]	−1.50 x10^−3^ ±5.50 x10^−3^ (rel·yrs^−1^)	27
NRA-Global (mm^2^)	[45;79]	1.15 x10^−2^ ±1.40 x10^−2^ (mm^2^·yrs^−1^)	35
cpRNFLT-Global (μm)	[45;81]	−0.14 ±0.46 (μm·yrs^−1^)	36
GDx-TSNIT (μm)	[45;81]	−5.48 x10^−2^ ±22.0 x10^−2^ (μm·yrs^−1^)	34

### Estimation of sources of variation in measurements in not-glaucoma suspect eyes

Data for analysis were selected specifically for each quantity aiming for maximum precision (highest d.f.) in estimates of variance for iterations of measurement within occasion. For IOP and MD, respectively, data with the maximum number of non-suspect eyes with measurements at both the 1^st^ and the 2^nd^ occasion were selected. For the other variables, the maximum number of subjects with the largest possible number of iterations were identified for maximum precision in the analysis of variance. For this, the product of the available number of subjects at the 1^st^ and the 2^nd^ occasion, both with the same number of iterations available within occasion were considered.

For IOP and MD, the sources of variation in non-suspect eyes were estimated with an analysis of variance according to the model provided in Appendix 4. For the other quantities, the sources of variation were estimated with an analysis of variance according to the model provided in Appendix 5.

For IOP and MD, respectively, the variation among iterations is included in the estimated variance for occasions (Table 5) since measurement from only one iteration was recorded (Appendix 4, Eq. 2). For IOP and MD, the variance for occasions was estimated to approximately half of the variance for subjects.

**Table 5:** Estimated sources of variation for quantities suggested for evaluation of glaucoma progression

Quantity	Age interval	Estimated variance components						
		Unit	Subjects	n^1^	Occasions^2^	n^1^	Iterations	n^1^
IOP	[45;82]	mmHg^2^	3.77	40	2.14	2		
MD	[46;82]	dB^2^	2.18	37	1.01	2		
C/D-linear	[45;80]	rel.^2^	1.32 x10^−2^	25	22.8 x10^−6^	2	4.32 x10^−4^	4
NRA-Global cpRNFLT-Global^3^	[45;80]	mm^4^	14.8 x10^−2^	31	2.91 x10^−4^	2	21.0 x10^−4^	4
	[45;82]	μm^2^	153	36	6	2	6	3
Stratus cpRNFLT-Global	[45;82]	μm^2^	171	15	11	2	10	3
Cirrus cpRNFLT-Global	[53;80]	μm^2^	146	19	3	2	3	5
GDx-TSNIT	[45;80]	μm^2^	29.7	11	0.7	2	1.4	3

^1^number analyzed. Considerable variation due to variable subject cooperation and occasionally device malfunction at subject visit

^2^variance component occasions for IOP and MD contains variance component for iterations

^3^record represents Stratus and Cirrus measurements pooled together and separated into measurements with Stratus only and measurements with either Cirrus 4000 or Cirrus 5000.

For the HRT quantities, the variability among subjects was several orders of magnitude larger than the variability among occasions.

cpRNFLT-Global measurements with the Cirrus machine resulted in lower variabilities among subjects, occasions and iterations than with the Stratus machine (Table 5).

### Estimation of frequency distribution for non-suspect eyes measured at one occasion

For IOP and MD, the frequency distribution for measurements at baseline was estimated from the subset of non-suspect eyes fulfilling the criterion that at least one measurement was available at each of the 1^st^ and the 2^nd^ occasion (Table 5).

For C/D-linear, NRA-Global, cpRNFLT-Global and GDx-TSNIT, the frequency distribution for measurements at baseline in the subset of non-suspect eyes was estimated considering subjects with 3 iterated measurements available at the first occasion and averaging over the 3 iterations.

The frequency distributions for the quantities analyzed are shown in plots of Normal Equivalent Deviates (NED) as a function of magnitude of observation [13].

**Fig. 2:**
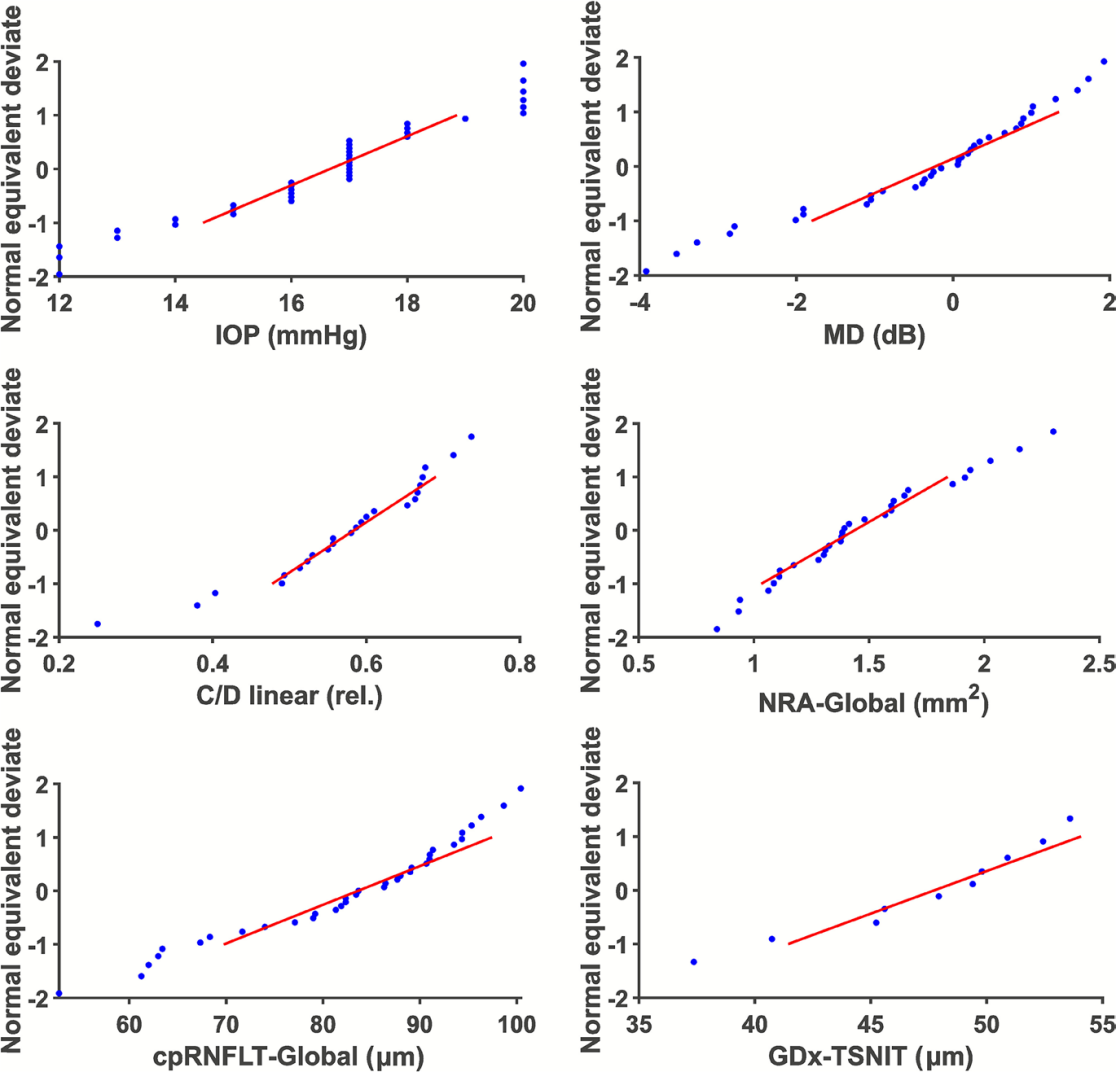
Normal Equivalent Deviate versus observation for observed quantities. The red line is a linear regression of the observations within 1 standard deviation (0.68 of the all potential observations) considering a first order relationship

For all observed quantities, the observations within 1 standard deviation around the mean appears to be fit a normal distribution (Fig. 2).

### Estimation of a confidence limit for a one sided tolerance limit

A confidence limit for a one sided tolerance limit was estimated according to Appendix 1.

Normal distribution parameters for the quantities analyzed were estimated based on 1 iteration within occasion for IOP and MD and average of 3 iterations within occasion for C/D-linear, NRA-Global, cpRNFLT-Global and GDx-TSNIT (Table 6).

**Table 6:** Estimated normal distribution parameters and corresponding confidence intervals for extreme tolerance limit

Quantity	$\overline{x }$	s	D.f	CI_CV_(0.95) (Rel.)	Critical limit estimated		
					CI_Tolerance(0.95)_(0.95)	Limit in relation to mean	Critical limit distance from mean normalized to mean
IOP (mmHg)	17	2	39	[0.11;0.18]	22	Above	0.30
MD (dB)	−0.33	1.6	36	*	-3.7	Below	
C/D-linear (rel.)	0.58	0.12	24	[0.15;0.27]	0.84	Above	0.46
NRA-Global (mm^2^)	1.5	0.4	30	[0.20;0.34]	0.6	Below	0.6
cpRNFLT-Global (μm)	83	12	35	[0.12;0.19]	56	Below	0.32
Stratus cpRNFLT-Global (μm)	82	13	14	[0.12;0.25]	48	Below	0.41
Cirrus cpRNFLT-Global (μm)	84	12	18	[0.11;0.21]	55	Below	0.35
GDx-TSNIT (μm)	48	5	10	[0.08;0.20]	33	Below	0.31

^*^CV and critical limit relative change from mean not estimated since expected mean = 0

CI_Tolerance(0.95)_(0.95) estimated based on μ = 0 and upper confidence limit for s.

Considering that only small samples were available, confidence limits for coefficient of variation were estimated [35]. Further, a confidence limit for the relevant extreme tolerance limit was estimated [14] in the direction expected for glaucoma (Table 6, 6th column). The relevant extreme tolerance limit is below the mean for quantities that are expected to decrease due to glaucoma and above the mean for quantities that are expected to increase due to glaucoma. For MD measurements, the expected mean is 0. Therefore, the confidence limits for the tolerance limit was based on the confidence limits for the estimated standard deviation, considering a normal distribution with μ=0.

**Fig. 3:**
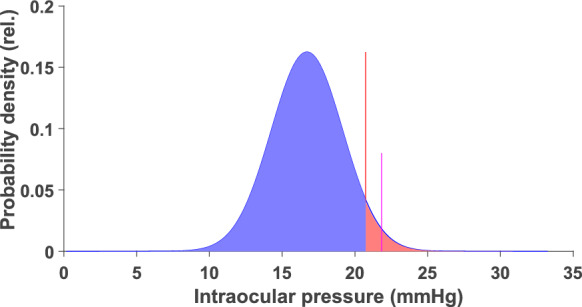
The critical limit estimated as the extreme 95 % confidence limit (vertical purple line) for the upper 95 % tolerance limit (vertical red line) for evaluation of the first IOP measurement in a potential glaucoma patient derived from the estimated mean and standard deviation in the present study. Blue surface: Probability for IOP in a not glaucoma suspect subject below the upper tolerance limit assuming a normal distribution. Red surface: Probability for IOP above the tolerance limit that will be wrongly classified as pathological although the eye is not glaucoma suspect

The coefficient of variation, CV (Table 6, 5th column), indirectly measures the absolute difference between the critical limit from the expected mean in relation to the expected mean. A high CV reflects a widespread distribution, and therefore a large distance between the critical limit and the expected mean (Fig. 3). Normalizing the critical limit distance from the mean to the mean (Table 6, last column) allows comparison of the efficiency for sensitivity estimation among quantities. The high relative numbers for C/D-linear and NRA-Global thus indicate lower efficiency for sensitivity estimation than the other quantities.

Although the sample sizes are small, the HRT quantities appear to require a larger deviation for classification as outside the critical limit than the other quantities. The outcome implicates that IOP, MD, cpRNFLT-Global and GDx-TSNIT require deviations of the same order for classification as outside the critical limit. For all quantities measured holds that when comparing a measurement to a critical limit defined as a tolerance limit for measurements in subjects, resolution for detection of deviation from normality is limited by the variability among subjects (Table 5).

## Discussion

Several quantities have been proposed for quantitative assessment of glaucoma. The present study intended to estimate critical limits for not pathological eyes that can be used to classify eyes as glaucomatous in the population sampled. Such critical limits are necessary for estimation of the relative sensitivity for detection of glaucoma for quantities estimated in the UGDS study. The limited sample size is associated with uncertainty and was considered by expressing the critical limit as an extreme confidence limit. Critical limits intended for clinical differentiation between glaucoma and not glaucoma should be derived from a sample size of at least 250.

In the present study, the non-glaucoma population sampled consisted of eyes clinically not considered glaucoma suspect in subjects where the fellow eye was clinically considered glaucoma suspect. Therefore, the critical limit estimated for differentiation between glaucoma and not glaucoma is valid only for initial clinical evaluation of eyes that may later convert to glaucoma.

The different quantities presently analyzed were often but not always measured in the same eye. All estimates are therefore dependent. The comparison of estimates is consequently limited to description. However, since all measurements for all quantities are related to the same cohort of subjects it is anticipated that discrepancies between the quantities express real differences.

The variation of sample size for different quantities was caused by variable subject cooperation, and occasional device malfunction requiring repair and re-calibration by the distributor (Table 5, footnote 1). The variation impacts the precision in estimates of mean and standard deviation and indirectly impacts the precision expressed in the confidence intervals for the variation coefficients and the tolerance limits.

The cpRNFLT-Global measurements were derived from 3 different generations of OCT devices representing technology steps that allow increasingly faster sampling. Increased sampling rate translates into increasing measurement precision since eye movement limits the precision within 1 ONH capture. In the current paper, analysis was made on the cpRNFLT-Global data collected with the 3 different OCT-devices pooled together and separated into data captured with time-domain and frequency domain, respectively (Table 6).

The scatterplots demonstrated that the presently analyzed sub-cohort of not glaucoma suspect eyes covered the age interval when glaucoma typically occurs and that both sexes were evenly distributed over the age interval (Fig. 1).

The lack of iterated measurements of IOP is a limitation of the data available. For this reason, the value of averaging over several IOP measurements at each occasion cannot be conclusively judged. The variance for subjects and occasions was of the same order (Table 5) and the variance for occasions estimated with the model in Appendix 2, Eq. 2, is the sum of variances for occasions and iterations. It is probable that iteration of IOP measurements would improve precision.

For the MD estimates, the estimated variance for subjects (Table 5) expresses the subject variability around the reference population used in the Humphrey machine since MD is age and sex adjusted to the reference population.

The sum of the variance for subjects and occasions was large for all the quantities analyzed (Table 5). At the first visit, a measurement necessarily has to be compared to a critical limit defined by the variance for subjects. Since the variance for subjects is substantial (Table 5) such an evaluation is inefficient.

For IOP, the precision can probably be increased by averaging over iterations. Also, for MD, precision would probably improve by averaging over several measurements at each occasion. However, this would be very cumbersome in clinical routine work due to the time consumption and generate unrealistic burden for the patient. For the other quantities averaging over three iterations would be possible in clinical routine work and is desirable to gain some precision (Appendix 3, Eq. 4).

Glaucoma is supposed to cause either an increase or a decrease depending on the quantity measured. Therefore, one sided tolerance limits were estimated. On the condition that the normal distribution is a good approximation, critical limits defined as tolerance limits may be defined based on a known expected population mean and standard deviation, respectively. However, in the current study the expected mean and standard deviation were estimated from a limited sample. Therefore, extreme confidence limits for one sided tolerance limits were estimated [14].

Typically, critical limits should be calibrated to age and sex. Our finding that the MD measurements are independent of age and sex is consistent with the fact that MD by definition is age and sex adjusted. Other studies have shown that cpRNFLT-Global decreases with age [1], [10], [11]. Considering the variability of cpRNFLT-Global among subjects the sample size in the current study was too small to resolve a cpRNFLT-Global loss with age (Fig. 1, Table 5). For the other quantities measured, it is similarly possible that the substantial variability among subjects (Table 5) obscure a slight age and or sex dependence.

Critical limit estimates based on the normal distribution requires data that are approximately normal distributed. The deviation from the normal distribution at extreme values in the NED plots (Fig. 2) is expected due to the limited sample size. Statistical verification of an underlying normal distribution would require a much larger sample than was available. The observations for all the quantities measured within 95% around the mean are close to normal distributed (Fig. 2). The comparison of critical limit distance from mean normalized to mean among quantities measured (Table 6, last column) is therefore relevant. The C/D-linear measurements are relative numbers between 0 and 1. The frequency distribution is therefore expected to be truncated close to the extreme values. However, the estimated mean C/D-linear was centered at 0.6 (Table 6). Variation around the center between the extreme limits is therefore expected to be approximately normal distributed. This was supported by the NED-plot for C/D-linear measurements (Fig. 2).

The precision of measurement of eye within subjects depends on variability among subjects, occasions within subjects, and measurements (Appendix 5). The analysis of variance demonstrated that the variance for subjects dominated for all quantities estimated (Table 5). This agrees with previous findings for NRA [32]. Consequently, the capacity to distinguish pathological from not pathological is, for all critical limits derived, limited by substantial variability among subjects. This implies that critical limits derived from population estimates of mean and standard deviation are insensitive for detection of pathological. Sensitivity would increase substantially if critical limits derived from within subjects measurements, i.e. multiple occasions, are applied.

The currently observed variabilities for IOP measurements (Table 5) were of the same order that have previously been published [8], 29. Several IOP measurements could easily be iterated in clinical routine work. Averaging would reduce the total variation for subjects (Eq. 4). The presently observed variabilities among MD measurements are comparable to previously published data (Russell, Garway-Heath & Crabb 2013). Averaging of several measurements at the same occasion is clinically impossible due to time consumption of the measurement procedure and inconvenience for the patient. The variability among subjects was larger for Stratus than Cirrus (Table 5). Previous estimates of precision of subject measurements are of the same order and found a similar difference [20], [27], [34]. The presently observed lower variabilities for cpRNFLT-Global measurements with the Cirrus device than for the Stratus device (Table 5) translates into shorter critical limit distance from the mean, normalized to the mean (Table 6). The here observed variabilities for the morphometric variables were approximately similar to previously published data: C/D-linear, NRA-Global, cpRNFLT-Global, GDx-TSNIT [9], [12], [25]. For the morphometric quantities measured, the variance for iterations was low in relation to the sum of the variance for subjects and occasions (Table 5). Therefore, averaging over several iterations at each occasion is expected to have only limited impact on measurements in subjects (Eq. 4).

In the current study, the estimated extreme confidence limit for the one sided 95% tolerance limit corresponds to a specificity of 95 % for the quantity indicated. The currently estimated critical limits for IOP, C/D-linear, and NRA-Global correspond to the previously published critical limits (Table 2). The lower-than-expected critical limits for GDx-TSNIT and cpRNFLT may arise from device-specific precision and calibration and be associated with differences in populations measured. These differences emphasize the importance of establishing clinical center based critical limits. A small sample size when estimating the confidence limit for the tolerance limit introduces uncertainty. Thus, a confidence limit for a small sample is further away from the mean than for a large sample. The small sample size available for GDx-TSNIT therefore contributes to the low estimated critical limit (Table 6, 6^th^ column) in comparison to previous data (Table 2, last row).

Not considering MD, the critical limits presently established, suggest that for glaucoma detection a larger relative change from expected mean in not glaucoma suspect eye is required for the HRT quantities measured than for the other quantities measured (Table 6, last column). This is consistent with previous estimations of variability [32].

It should be pointed out that a critical limit based on level and variability of a measured quantity defines the probability to wrongly classify a measurement as pathological although it is not (1 minus the tolerance selected, where tolerance is equivalent to specificity). The clinical significance of the critical limit depends on the importance of a defined change with regard to the disease, in the present study glaucoma. If an IOP above the statistically defined critical limit does not impact the progress of the disease, measurements of IOP are not suitable for detecting glaucoma even if the critical limit is close to the expected mean in non-glaucoma eyes.

Critical limits for discrimination of pathology from non-pathology used at the initial patient visit necessarily includes variability among subjects and therefore are insensitive for detection of glaucoma. The current analysis indicates similar but better potential capacity to discriminate glaucoma from not glaucoma at any arbitrarily defined specificity for IOP, cpRNFLT-Global and GDx-TSNIT than for HRT measurements at the initial patient visit (Table 6, last column).

To evaluate the capacity of a quantity to correctly identify glaucoma, the phenotype of glaucoma in focus has to be uniquely specified. Further, it must be demonstrated that the expected mean of the quantity analyzed is related to the grade of the phenotype of glaucoma in focus. Thus, any estimation of sensitivity for a specific phenotype of glaucoma depends on the definition of the phenotype.

It is concluded that it is preferable to estimate critical limit for small samples as extreme confidence limit for the tolerance limit. The presently estimated critical limits are comparable to previous findings for the device models used. C/D-linear, and NRA-Global estimated with HRT provides lower efficiency for estimating sensitivity than the other quantities measured. Specific intra-individual critical limits require a small increase of the measured quantity to identify glaucoma when the patient has glaucoma.

## Data Availability

No datasets were generated or analysed during the current study.

## Appendix 1: Estimation of a critical limit for classification of not glaucoma suspect eye

Measurements of a defined quantity on a not pathological object are expected to vary randomly. A critical limit for distinction of pathological from not pathological may then be defined as the measurement level that corresponds to a chosen probability for correct classification as not pathological. The chosen probability is often referred to as specificity. The specificity thus defines the probability for a measurement inside the critical limit (higher than the critical limit if pathology reflects reduction and lower than the critical limit if pathology reflects increase).

On the condition that the frequency distribution for not pathological measurements of a response variable can be considered normal distributed and the population mean and standard deviation are known, a critical limit for distinguishing pathological from not pathological can be defined as a one-sided tolerance level (Fig. 3, vertical red line). The tolerance then is equivalent to the specificity.

Estimation of mean and the standard deviation of the not pathological population from a limited sample introduces an uncertainty in the one sided tolerance level that can be estimated as confidence limits for the one sided tolerance limit [14]. The critical limit is then the extreme confidence limit of the one sided tolerance limit (Fig. 3, vertical purple line).

## Appendix 2: Inclusion criteria in the Uppsala Glaucoma Detection Study

- >18 yrs
- Open anterior chamber angle in both eyes.
- Absence of media opacities that obstruct imaging of the optic nerve head.
- Clear ophthalmoscopic demarcation of the optic nerve head and surrounding tissue.
- No use of pressure-reducing medication before inclusion.
- At least one glaucoma suspect eye.An eye is glaucoma suspect if at least one of the below criteria is fulfilled:
  - Glaucoma hemifield asymmetry as judged by an experienced glaucoma specialist at visual inspection of the Humphrey visual field pattern deviation.
  - Ocular hypertension (intraocular pressure > 21 mmHg).
  - Intraocular pressure asymmetry (one eye > 5 mmHg different from the other).
  - Pseudo-exfoliation
  - Pigment dispersion syndrome
- Ability to undergo computerized visual field testing.
- Willingness to participate in follow-up assessments every 4 months for 5 years.
- Capacity to comprehend and provide informed oral and written consent.

## Appendix 3: Regression model for analysis of an effect of age and sex on the outcome

The quantity, *q* (quantity specific unit), is the sum of a term for the quantity at age 0 years, *q*_*0*_ (quantity specific unit), a term consisting of a factor for age, *A* (years), and a factor for change of the quantity per year, *k* (quantity specific unit·yrs^−1^), a term consisting of a factor for sex, *S* coded as a dummy variable (Male=1/Female=0) and a factor expressing the sex dependence, *k*_*S*_ (yrs^−1^) , a term consisting of a factor for the interaction between age and sex, *AxS* (yrs) and a factor expressing the impact of the interaction factor, *k*_*Axs*_ (quantity specific unit·yrs^−1^), and a term for random error, *ε* (Eq. 1).

1 $${\mathrm{q}} = {\text{ q}}_{0} + {\mathrm{k*A}} + {\mathrm{k}}_{{\mathrm{s}}} {\mathrm{*S}} + {\mathrm{k}}_{{{\mathrm{AxS}}}} {\mathrm{A*S}} + {\upvarepsilon }$$

First, the full 5 term model was fitted and the residual variance was to be estimated. Then, a reduced model omitting the interaction term, k_Axs_·A*S, and estimating the residual variance was applied. The significance of the interaction term was then tested by comparing the residual variance for the reduced 4 term model to the residual variance with the full model with an F-test. If the interaction term was found to be insignificant for the residual variance, it was planned to compare the 3 term model (omitting the interaction term and the term for sex dependence) to the residual variance for the 4 term model with an F-test. If the term for the impact of sex, *k*_*S*_* S*, was found to be insignificant for the residual variance, the 3 term model was to be fitted to estimate the dependence on age only as a confidence interval for the age dependence, *k*.

## Appendix 4: Analysis of variance model, quantities without iteration at occasion

The variation among subjects and between the first and the second occasion is analyzed according to the model in Eq. 2.

2 $${\mathrm{x}}_{{{\mathrm{ij}}}} = {\mathrm{A}}_{{\mathrm{i}}} + {\upvarepsilon }_{{{\mathrm{j}}\left( {\mathrm{i}} \right)}}$$

Each measurement, *x*_*ij*_*,* is the sum of the population mean, μ, and a term for the random variation among subjects, *A*_*i*_ (i=1,2..*a*: number of subjects) and a term for the random variation of occasions and measurement error, ε_j(i)_ (j=1,n: number of iterations at each occasion).

The analysis of variance provides estimates of $\sigma_{A}^{2}$ with *a-1* degrees of freedom and $\sigma_{\varepsilon }^{2}$ with *a*(n-1)* degrees of freedom.

## Appendix 5: Analysis of variance model, quantities with iteration at occasion

The variation between the first and the second occasion is analyzed according to the model in Eq. 3.

3 $${\mathrm{x}}_{{{\mathrm{ijk}}}} = {\mathrm{A}}_{{\mathrm{i}}} + {\mathrm{B}}_{{{\mathrm{j}}\left( {\mathrm{i}} \right)}} + {\upvarepsilon }_{{{\mathrm{k}}\left( {{\mathrm{ij}}} \right)}}$$

Each measurement, *x*_*ijk*_*,* is the sum of the population mean, *μ*, and a term for the random variation among subjects, *A*_*i*_ (i=1,2,..*a*: number of subjects), a term for the random variation among occasions, *B*_*j(i)*_ (j=1,2..*b*: number of occasions in each subject), and measurement error, *ε*_*k(ij)*_ (k=1,2..*n*: number of iterations at each occasion).

The analysis of variance provides estimates of $\sigma_{A}^{2}$, $\sigma_{B}^{2}$ and $\sigma_{\varepsilon }^{2}$, the last with *a***b**(*n*-1) degrees of freedom.

Considering measurements, *x*, obtained by averaging *n* iterations within each occasion, the variance, $\sigma_{x}^{2}$ depends on the variance for subjects, the variance for occasion and the variance for iterations (Eq. 4).

4 $${\upsigma }_{{\mathrm{x}}}^{2} = {\upsigma }_{{\mathrm{A}}}^{2} + {\upsigma }_{{\mathrm{B}}}^{2} + \frac{{{\upsigma }_{{\upvarepsilon }}^{2} }}{{\mathrm{n}}}$$
